# NGS-секвенирование в селекционно-генетических исследованиях ячменя

**DOI:** 10.18699/VJ20.627

**Published:** 2020-07

**Authors:** I.V. Rozanova, E.K. Khlestkina

**Affiliations:** Federal Research Center the N.I. Vavilov All-Russian Institute of Plant Genetic Resources (VIR), St. Petersburg, Russia Institute of Cytology and Genetics of Siberian Branch of the Russian Academy of Sciences, Novosibirsk, Russia; Federal Research Center the N.I. Vavilov All-Russian Institute of Plant Genetic Resources (VIR), St. Petersburg, Russia Institute of Cytology and Genetics of Siberian Branch of the Russian Academy of Sciences, Novosibirsk, Russia

**Keywords:** Hordeum vulgare, SNP, barley, genome, sequencing, throughput genotyping, genomic editing., Hordeum vulgare, SNP, ячмень, геном, секвенирование, высокопроизводительное генотипирование, геномное редактирование.

## Abstract

Ячмень (Hordeum vulgare L.) – один из важнейших видов злаковых растений, используемых в качестве продовольственной и кормовой культуры, а также для пивоварения и производства спирта. В конце прошлого столетия к традиционным методам селекции прибавились методы, основанные на применении ДНКмаркеров. Молекулярные маркеры также активно вовлекаются в процессы молекулярно-генетического картирования и анализа QTL (quantitative trait loci). В 2012 г. было завершено секвенирование генома ячменя, что выявило целый спектр новых возможностей – от более эффективного поиска генов-кандидатов хозяйственно ценных признаков до геномной селекции. В обзоре обобщены результаты работ периода после секвенирования генома ячменя, открывшего новые направления генетики и селекции этой культуры с применением высокопроизводительных методов секвенирования и генотипирования. В рассматриваемый период ведутся интенсивные исследования по идентификации геномных локусов ячменя, ассоциированных с хозяйственно ценными признаками, появились и пополняются ресурсы для работы с геномными данными ячменя и для их депонирования. В последние годы для массового поиска ассоциаций между фенотипом и генотипом используется анализ GWAS (genome wide association studies), широкое применение которого на ячмене стало возможным с 2010 г. благодаря разработанным SNP-чипам, а также методам генотипирования, основанным на прямом NGS-секвенировании (next generation sequencing) выборочных фракций генома. К настоящему времени опубликовано более 80 работ, описывающих результаты GWAS-анализа на ячмене. Идентификация SNP, ассоциированных с хозяйственно ценными признаками, и их преобразование в удобные для скрининга селекционного материала CAPS или KASP-маркеры существенно расширяют возможности маркер-ориентированной селекции ячменя. Кроме того, имеющаяся информация о потенциальных генах-мишенях и качество полногеномной последовательности ячменя представляют достаточную базу для применения технологий геномного редактирования с целью создания исходного материала для селекции сортов с заданными свойствами.

## Abstract (en)

Barley (Hordeum vulgare L.) is the one of the most important cereal species used as food and feed crops, as well as for malting and alcohol production. At the end of the last century, traditional breeding techniques were complemented by the use of DNA markers. Molecular markers have also been used extensively for molecular genetic mapping and QTL analysis. In 2012, the barley genome sequencing was completed, which provided a broad range of new opportunities – from a more efficient search for candidate genes controlling economically important traits to genomic selection. The review summarizes the results of the studies performed after barley genome sequencing, which discovered new areas of barley genetics and breeding with high throughput screening and genotyping methods. During this period, intensive studies aimed at identification of barley genomic loci associated with economically important traits have been carried out; online databases and tools for working with barley genomic data and their deposition have appeared and are being replenished. In recent years, GWAS analysis has been used for large-scale phenotypegenotype association studies, which has been widely used in barley since 2010 due to the developed SNP-arrays, as well as genotyping methods based on direct NGS sequencing of selected fractions of the genome. To date, more than 80 papers have been published that describe the results of the GWAS analysis in barley. SNP identification associated with economically important traits and their transformation into CAPS or KASP markers convenient for screening selection material significantly expands the possibilities of marker-assisted selection of barley. In addition, the currently available information on potential target genes and the quality of the whole barley genome sequence provides a good base for applying genome editing technologies to create material for the creation of varieties with desired properties.

## Введение

Ячмень (Hordeum vulgare L.) – одна из важнейших сельскохозяйственных культур, занимающая пятое место в мире после пшеницы, кукурузы, риса и сои по площади посевов (47 млн га), согласно данным 2017 г. (http:// www.fao.org/faostat/ru/). Зерно ячменя идет в основном на кормопроизводство (до двух третей от всего урожая), пивоваренную промышленность и производство спирта (около одной трети) и лишь небольшой процент приходится на долю продовольственного, т. е. применяемого для пищи человека (в Российской Федерации, по данным 2017 г., – 0.12 %, http://www.fao.org/faostat/ru/). Кроме того, отмечен рост значимости ячменя как источника сырья для производства крахмала и крахмалопродуктов пищевого и технического назначения (Blennow et al., 2013).

По сравнению с основным хлебным злаком – пшеницей – ячмень неприхотлив, легче адаптируется к неблагоприятным условиям окружающей среды – холоду, засухе, лучше переносит защелачивание и засоление почвы, недостаток питательных веществ в ней. Раннее созревание в сочетании с высокой адаптивностью позволили ячменю как сельскохозяйственной культуре распространиться по всему миру – от экватора до северных и южных широт – более чем в 100 странах мира (http://www.fao.org/faostat/ru/). Благодаря ряду биологических особенностей ячменя (относительно короткий жизненный цикл, самоопыление, диплоидный геном) современные молекулярно-генетические и геномные исследования этой культуры продвигаются динамичнее, чем в случае других представителей Triticeae, в частности пшеницы и ржи (Hayes, Szucs, 2006; Schulte et al., 2009). Вследствие этого ячмень служит модельным растением, а полученные данные о его генах и геноме используются для продолжения генетических исследований на других представителях трибы Triticeae.

Развитие методов NGS-секвенирования позволило получить качественные полногеномные последовательности многих видов растений (Брагина и др., 2019), включая ячмень (International Barley Genome Sequencing Consortium, 2012) и пшеницу (Appels et al., 2018). Секвенирование геномов, а вслед за этим секвенирование транскриптомов и микроРНКомов вывели на новый этап исследования о функционировании наследственного материала. Получаемые сведения применяются для маркер-контролируемого отбора в селекционном процессе, а также для разработки принципиально новых технологий создания исходного селекционного материала с заданными свойствами на основе геномного редактирования (Хлесткина, Шумный, 2016; Korotkova et al., 2019)

Цель настоящего обзора – обобщить результаты генетико-селекционных работ, базирующихся на применении методов NGS-секвенирования.

## Секвенирование генома ячменя и его структурная организация

Для секвенирования генома ячменя в 2006 г. был сформирован Международный консорциум (International Barley Genome Sequencing Consortium…, 2012), который изначально включал в себя исследователей из восьми научных организаций шести стран: Германии, США, Австралии, Японии, Финляндии и Шотландии. Позднее к консорциуму присоединились группы ученых из Великобритании, Израиля, Франции и Италии. 

К 2009 г. был собран первый вариант последовательности генома ячменя (сорт Morex), которая была необходима для дальнейшего секвенирования генома, но вместе с тем оказалась полезной и широкому кругу исследователей, нацеленных на выделение отдельных генов, контролирующих хозяйственно ценные признаки (Schulte et al., 2009). Основу физической карты составили более 83 тыс. ген-содержащих BAC-клонов (bacterial artificial chromosome, бактериальных искусственных хромосом), которые исследовались методом гибридизации с применением избыточных зондов (метод overgo) (Madishetty et al., 2007). Данные были получены с помощью высокопроизводительного фингерпринтинга (fingerprinting) (Luo et al., 2003)

В 2012 г. создана референсная карта генома ячменя (International Barley Genome Sequencing Consortium…, 2012). Для этого были секвенированы и проанализированы последовательности 571 тыс. ген-содержащих ВАСклонов из шести независимых геномных библиотек

В дальнейшем работы по уточнению полногеномной физической карты были продолжены. В частности, использован метод МТР (minimum tiling path), основанный на принципе минимального количества составных частей карты при максимальной плотности покрытия (Ariyadasa et al., 2014). Кроме того, разработана ультраплотная генетическая карта. Для этого было проведено генотипирование на основе высокопроизводительного секвенирования потомства из 90 рекомбинантных инбредных линий (RILs) от скрещивания Morex*Barke (M×B) и 82 дигаплоидных линий (DH) из картирующей популяции Oregon Wolfe Barley (Cistué et al., 2011). Сконструированные генетические карты были соотнесены с физической картой сорта Morex, полученной в результате работы Консорциума (Mascher et al., 2013). Таким образом, генетические локусы были сопоставлены с положением на физической карте. Суммарная длина прочитанного и собранного генома ячменя составила 4.98 Гб – более 95 % генома, если учесть, что, согласно имеющимся оценкам, 100 % – это 5.1 Гб. 

Полученный материал позволил создать ресурсы (Приложение 1)^1^, ценные для дальнейших исследований, на правленных на изучение функциональной организации генов и генома ячменя, и для прикладных работ, нацеленных на выведение новых улучшенных сортов, в том числе с применением методов селекции следующего поколения (next-generation breeding), а именно систем генетического редактирования. 

 Приложения 1 и 2 см. по адресу: http://www.bionet.nsc.ru/vogis/download/pict-2020-24/appx4.pdf



## Структурная организация генома ячменя

Согласно распределению уникальных последовательностей и повторов в хромосомах ячменя, выделяют три зоны. Зона 1 – дистальная – характеризуется большим количеством низкокопийных элементов (low-copy regions, LCRs), высоким содержанием генов и высокой частотой мейотических рекомбинаций. Зона 2, занимающая промежуточное положение на хромосомах, обладает средней плотностью генов. В третьей зоне, проксимальной, количество генов минимальное (Mascher et al., 2017). 

Выделенные зоны различаются не только по количеству генов, но и по их специализации. Так, в проксимальных (прицентромерных) участках хромосом, расположены главным образом «гены домашнего хозяйства» – консервативные гены, важные для жизнедеятельности клетки, мутации по которым, как правило, летальные. Эта фракция генома не является материалом для искусственного отбора. Гены, потенциально интересные для селекционеров, например участвующие в защитном ответе и репродуктивных процессах, расположены преимущественно в дистальных районах (концевые участки хромосом), которые, в противоположность проксимальным, характеризуются высокой частотой рекомбинации (Mascher et al., 2017).

Большая часть генома растений состоит в основном из очень похожих копий повторяющихся элементов, таких как ДНК-транспозоны (MITE, miniature inverted-repeat transposable elements, CACTA-элементы) и ретротранспозоны, включая LTR (long terminal repeats – ретротранспозоны с длинными концевыми повторами) и LINE (long interspersed nuclear element – подтип с длинными диспергированными повторами). В настоящее время считается, что эти элементы играют значительную роль в функциональной организации и эволюции геномов растений. Сравнение с библиотекой повторов, специфичной для Triticeae, выявило, что 3.7 Гб (80.8 %) от собранного генома ячменя относится к мобильным элементам, причем только 10 % этих элементов потенциально активны (Mascher et al., 2017). Повторы MITEs и LINEs в основном представлены в богатых генами дистальных районах в зоне 1. Локализация LTR-ретротранспозонов зависит от их типа: Ty3-Gypsy-ретротранспозоны обнаружены в зоне 3, тогда как Ty1-Copia преимущественно располагаются в зонах 1 и 2. 

Для генома ячменя характерно распределение разных мобильных элементов в зависимости от положения относительно генов. Мобильные элементы небольшого размера (например, транспозоны Mariner, относящиеся к семейству MITE) предпочтительно находятся в пределах 1 Кб, фланкируя кодирующие области генов, в то время как мобильные элементы более крупного размера (например, Harbinger, второе семейство MITE и LINEs) находятся на значительно более удаленном расстоянии от генов. Полученное распределение различных типов мобильных элементов среди генов может отражать давление отбора, которое позволяет только небольшим элементам, а именно транспозонам Mariners, находиться ближе всего к генам (Mascher et al., 2017). На большем расстоянии от генов доминируют мобильные элементы большого размера, такие как LTR-ретротранспозоны и транспозоны семейства CACTA.

## Пространственная организация генома ячменя

Полученная референсная последовательность генома ячменя также позволила исследовать пространственную организацию хроматина в ядре при помощи метода Hi-C (Mascher et al., 2017). Результаты подтвердили заключения, сделанные на основе более ранних исследований с применением методов микроскопии (Щапова, 1971; Künzel et al., 2000), о том, что хромосомы в ядрах ячменя принимают положение, известное в литературе под термином “Rabl orientation” (Cowan et al., 2001). Это конфигурация, при которой каждая хромосома в интерфазном ядре занимает место, во многом задаваемое позицией в анафазе предшествующего митоза. При этом хромосомы располагаются следующим образом: центромеры хромосом находятся на одном полюсе ядра, а теломеры ориентированы к противоположному. 

После основополагающей работы А.И. Щаповой (1971) было уточнено, что описанная конфигурация сохраняется у таких видов растений, как ячмень, пшеница, рожь и овес, во всех клетках растения (Anamthawat-Jónsson, HeslopHarrison, 1990), у риса – только в клетках ксилемы корня и в недифференцированных клетках пыльника (Prieto et al., 2004), у кукурузы и сорго подобной конфигурации хромосом не обнаружено (Dong, Jiang, 1998). 

Ранее было также установлено (Щапова, 1971), что ячмень обладает сходными по размеру плечами хромосом, тогда как у большинства видов эукариот все плечи по длине различаются и местоположение каждой хромосомы определено размерами ее плеч. Благодаря поляризации хромосомы складываются, как книжка, сопоставляя длинные и короткие плечи, в результате чего короткое плечо одной хромосомы имеет возможность взаимодействовать с локусами, расположенными на относительно таком же расстоянии от центромеры на длинном плече. Была отмечена также более высокая частота контактов между прителомерными регионами. При этом не имеет значения, гомологичны хромосомы или нет, контакты между материнcкой и отцовской хромосомами имеют место так же часто, как и между негомологами (Mascher et al., 2017). 

## Методы NGS-секвенирования для анализа полиморфизма ДНК как инструмента повышения эффективности маркер-ориентированной селекции

В основе картирования и маркирования генов и идентификации различных аллельных вариантов и в разработке технологий ускоренного и направленного отбора селекционного материала (маркер-ориентированная и геномная селекция) лежит анализ полиморфизма ДНК. Методы анализа полиморфизма ДНК «эволюционировали» от трудоемких подходов, основанных на блот-гибридизации (например, restriction fragment length polymorphism, RFLP – полиморфизм длины рестрикционных фрагментов) (Botstein et al., 1980) до гораздо менее трудоемких методов, основанных на полимеразной цепной реакции (ПЦР). Среди последних можно выделить такие ДНК-маркеры, как SSR – simple sequence repeats – простые повторяющиеся последовательности (Tautz, Renz 1984), STS – sequence tagged site – последовательности, характеризующие локус (Olson et al., 1989), и другие (Хлесткина, 2011). Позже появились высокопроизводительные методы с возможностью полной автоматизации процесса, такие как анализ SNP – single-nucleotide polymorphism – однонуклеотидный полиморфизм (Wang et al., 1998). Выявление SNP – результат работ по секвенированию и ресеквенированию сначала отдельных фракций, а затем и полных геномов

Появление, быстрое развитие и удешевление технологий секвенирования нового поколения (next generation sequencing, NGS) не только позволили секвенировать один за другим геномы хозяйственно значимых видов растений и животных (Хлесткина, 2013; Брагина и др., 2019), но и сделали возможными быстрое определение полиморфизма тысяч генов и разработку SNP-чипов для анализа генетического и селекционного материала. Для выявления SNP достаточно ресеквенировать небольшую выборку образцов. Полученные данные могут быть использованы для разработки SNP-чипов, пригодных к генотипированию больших выборок растений в относительно экономном режиме с большой пропускной способностью анализа. 

Позже на основе NGS-секвенирования были разработаны методы высокопроизводительного генотипирования, основанные на прямом секвенировании выборочных фракций генома изучаемых образцов. Один из них – метод GBS (genotyping-by-sequencing), впервые описанный в 2011 г. (Elshire et al., 2011), был успешно применен для генотипирования 276 рекомбинантных инбредных линий кукурузы и 43 дигаплоидных линий ячменя из картирующей популяции Oregon Wolfe Barley. В настоящее время термин “GBS” используется уже как обобщающий для различных разрабатываемых методов высокопроизводительного генотипирования, основанного на NGS-секвенировании (Rasheed et al., 2017). Геномная ДНК при этом обрабатывается эндонуклеазами рестрикции, далее создается библиотека фрагментов, при секвенировании их получают короткие прочтения (~100 пар нуклеотидов), объединяемые в контиги, выравнивание которых позволяет обнаружить SNP (Davey et al., 2011). Преимущество метода заключается в том, что его можно применять для анализа образцов не только видов с уже расшифрованным геномом, но и менее изученных видов, для которых еще не выполнена полногеномная сборка. Похожий на GBS метод RAD-seq (restriction-site-associated DNA sequencing), схожий в основе с методом GBS (используется подобный протокол секвенирования, основанный на присутствии в геноме сайтов узнавания эндонуклеазами рестрикции), но отличающийся протоколом подготовки библиотек (Chutimanitsakun et al., 2011; Andrews et al., 2016).

Метод GBS применяется для выявления взаимосвязи между фенотипом и генотипом на основе анализа двуродительских картирующих популяций (QTL-анализ – quantitative trait loci) или выборок сортообразцов (GWAS-анализ – genome wide association studies). Выявленные в результате этих работ геномные районы, ассоциированные с проявлением того или иного селекционно значимого свойства, сразу маркированы SNP-маркером. Если геном изучаемого вида уже секвенирован, то информация об обнаруженном районе генома, ассоциированного с признаком, может быстро привлекаться для поиска гена-кандидата, влияющего на изменчивость по данному признаку. Метод GBS только начинает применяться на ячмене (Fan et al., 2017; Darrier et al., 2019; Goddard et al., 2019), а результаты использования SNP-чипов уже применяются более 10 лет

Высокопроизводительное генотипирование ячменя впервые апробировано в 2005 г. (Rostoks et al., 2005) на основе технологии Illumina GoldenGate (Fan et al., 2003). Первые коммерчески доступные чипы для генотипирования ячменя появились в 2009 г. (Close et al., 2009). Для его создания было выбрано и протестировано 4596 SNP на 576 ДНК-образцах для двух пилотных чипов ОРА, РОРА1 и РОРА2, и на 480 ДНК-образцах для третьего пилотного чипа – РОРА3. Далее было выбрано 3072 SNP, которые прошли качественный контроль и были генетически информативны. Они составили две производные OPA – BOPA1 и BOPA2, которые планировалось использовать для дальнейших исследований генофонда ячменя. Из 3072 SNP, выбранных для изучения, 2279 были получены из библиотек EST, а 793 – путем секвенирования ПЦРампликонов. В результате в платформу для генотипирования, содержащую 3072 маркера, вошли два чипа, BOPA1 и BOPA2, каждый из которых содержал по 1536 маркеров SNP, и они имели различные дизайны генотипирования (Close et al., 2009). Следующий чип был разработан Illumina Infinium iSelect 9К Custom Genotyping BeadChip (Comadran et al., 2012) и включал в себя 2832 маркера, разработанных для предыдущей технологии GoldenGate, и 5010 дополнительных маркеров, основанных на обнаружении маркеров SNP в данных Illumina RNA-seq из 10 элитных сортов Великобритании (Bayer et al., 2017).

По мере снижения затрат на секвенирование постоянно росло число обнаруживаемых SNP. Поэтому на следующем этапе платформа 9K Infinium iSelect была расширена до 50K. Чип 50K включил около 6000 SNP из предыдущего чипа 9K и новые SNP, выявленные для ячменя на основе захвата экзома. Это эффективный метод, позволяющий проводить исследования генома по выбранным локусам, которые с большей вероятностью будут содержать полиморфизмы, сцепленные с интересуемыми признаками фенотипа (https://ics.hutton.ac.uk/50k; Bayer et al., 2017). 

## Методы анализа ассоциаций между геномными вариантами и фенотипическими признаками: QTL-анализ и GWAS

Первым методом поиска взаимосвязи генотипа с фенотипом, получившим широкое распространение, стал QTLанализ, или метод анализа локусов количественных признаков при использовании двуродительских популяций. При этом для изучения признака исследуется популяция потомства от контрастных по признаку родителей (Kearsey, Farquhar, 1998). Такой анализ на ячмене осуществляется с 1989 г. (Jensen, 1989), для него применялись различные типы маркеров, начиная с RFLP. В 2003–2010 гг. преобладали исследования, в которых QTL-анализ двуродительских популяций ячменя проводился на основе данных генотипирования, полученных при помощи SSRмаркеров. С 2010 г. для QTL-анализа ячменя преимущественно применялись популяции, генотипированные с помощью SNP-маркеров. С 2014 г. начали использоваться методы прямого секвенирования: GBS и RAD (Andrews et al., 2016; Darrier et al., 2019). Картирование значимых локусов с применением QTL-анализа дает высокую статистическую значимость для определяемого QTL, но в то же время имеет низкое разрешение. Существенный недостаток метода – ограничение генетического разнообразия родителей выбранной популяции (Hyne, Kearsey, 1995).

В последние годы для массового поиска ассоциаций между фенотипом и генотипом используется GWAS-анализ. На ячмене он применяется с 2010 г. (Lorenz et al., 2010). Сравнительный анализ работ показывает, что на основе GWAS обнаруживается больше новых локусов, по сравнению с QTL-анализом двуродительских популяций (Pauli et al., 2014). Преимущество метода заключается в высоком разрешении, вплоть до одного нуклеотида. Ассоциированный с признаком аллель детектируется частотой его наличия в популяции. Однако в этом случае можно упустить редкий аллель, так как значимость определения локуса будет зависеть от той же частоты аллеля. Для выявления локусов методом GWAS изучаемая популяция должна быть как можно более разнородна. Метод чувствителен к структуре популяции. Неправильно сформированная популяция может привести к установлению ложноположительных ассоциаций

Метод GWAS – достаточно новый, и, как видно по данным анализа публикаций, индексируемых в базе данных Scopus (рис. 1), в течение последних лет количество работ, выполненных с применением GWAS на ячмене, неуклонно возрастает. В настоящее время благодаря этому подходу обнаружены геномные районы, ассоциированные со многими признаками ячменя. Для нахождения компонентов урожайности и качества зерна ячменя с помощью GWAS изучаются агрономические признаки: высота растения, длина колоса, количество зерен в колосе, масса 1000 зерен, количество продуктивных побегов. Существует ~40 работ по исследованиям с использованием GWAS устойчивости ячменя к различным болезням и ~25 работ – по устойчивости к абиотическому стрессу – засухоустойчивости, устойчивости к засолению и др. Помимо этого, выполнены работы по изучению свойств крахмала, содержания белка, β-глюкана и микроэлементов в зерне. (Более подробно обзор работ представлен в Приложении 2.)

**Fig. 1.**
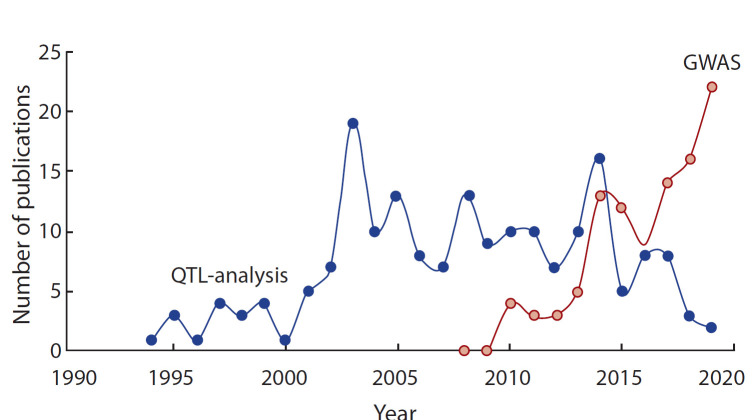
The numbers of publications on QTL and GWAS in barley revealed by queries “barley + genome-wide-association” and “barley + QTL-analysis” in the Scopus database (Website: http://scopus.com as to August 28, 2019).

Таким образом, для метода GWAS чаще всего используются SNP-чипы; SSR и DArT применяются редко, они были актуальны до разработки SNP-чипов и GBS (DArT – это, по сути, аналог GBS, но с применением чипов, а не прямого секвенирования). Метод GBS только начинает применяться для анализа полиморфизмов ДНК ячменя, ожидается, что он получит дальнейшее распространение. 

Как можно судить по публикациям, индексируемым в базе данных Scopus (рис. 2), работы по GWAS-анализу в равной мере направлены на проблемы устойчивости к фитопатогенам и вредителям (биотический стресс), толерантности к неблагоприятным абиотическим факторам окружающей среды и на повышение продуктивности растений.

**Fig. 2.**
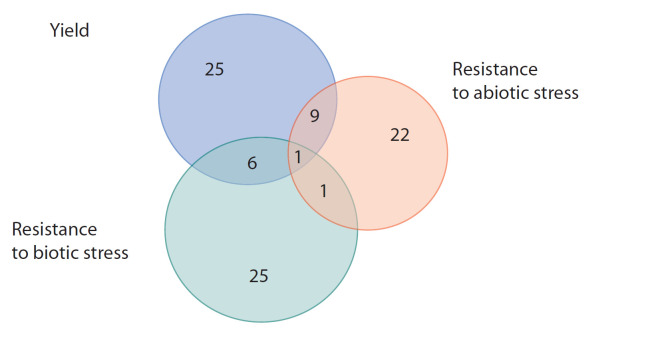
The genome-wide association (GWAS) in barley: number of publications revealed in the Scopus database as to August 29, 2019 by keyword intersection queries.

**Table 1.**
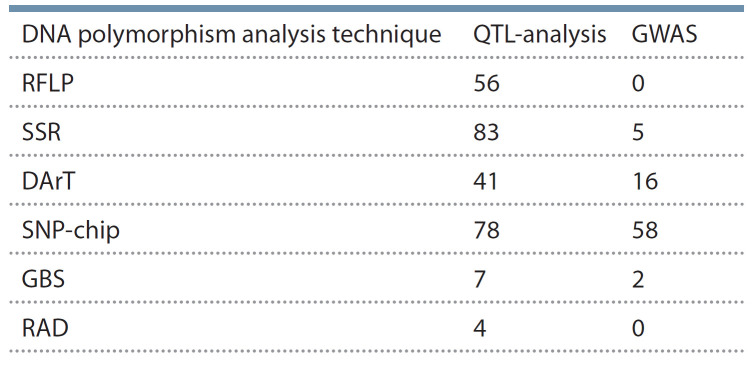
The summary on diff erent techniques of DNA polymorphism analysis and diff erent approaches (QTL and GWAS) to the identifi cation of loci associated with commercially important barley traits The summary on diff erent techniques of DNA polymorphism analysis and diff erent approaches (QTL and GWAS) to the identifi cation of loci associated with commercially important barley traits

Селекция на устойчивость позволяет сокращать поте- ри урожая, вызываемые фитопатогенами и вредителя- ми. В настоящее время бóльший успех наблюдается в идентификации генетических маркеров для признаков устойчивости к болезням, имеющих моно- или олигоген- ный контроль (ржавчинные болезни, головня, мучнистая роса). Сложнее идентифицировать генетические маркеры для устойчивости зерновых культур к фузариозу колоса, септориозам, гельминтоспориозным пятнистостям, кор- невым гнилям и другим болезням (Афанасенко, 2016). Часто уровень фенотипического проявления устойчивости зависит не от одного гена, а от суммарного эффекта всех генов устойчивости. Это ведет к постановке задачи перед исследователями – создать сорта с полигенной устойчи- востью, обеспечивающей средний уровень устойчивости, что выражается в замедленном развитии болезней. Эта задача отягощается тем, что возникают новые патотипы (Ghazvini, Tekauz, 2007; Leng et al., 2016).

Как использовать данные, полученные с помощью GWAS, в дальнейших программах селекции? Исследова- ния установленных геномных районов позволяют иденти- фицировать, валидировать и маркировать гены-кандида- ты. Можно и без поиска генов-кандидатов преобразовать выявленные SNP в удобные KASP- или CAPS-маркеры (Konieczny, Ausubel, 1993; Kumpatla et al., 2012; Semagn et al., 2014; Shavrukov, 2015), проверить их на независимых выборках и, при успешной валидации, рекомендовать применять эти маркеры для отбора селекционного материала. Этот подход может быть эффективен в случае локусов, существенно влияющих на изменение фенотипа. Полученная в результате GWAS-анализа информация о локусах с малым эффектом или локусах, эффект которых существенно зависит от генотипа, не будет играть роли для программ по маркер-ориентированной селекции, однако она является основой для развития работ по геномной селекции.

## Геномное редактирование

Наличие полногеномных последовательностей и известных последовательностей-мишеней для внесения задан- ных мутаций позволяет осуществлять редактирование целевых генов на основе систем ZFNs, TALENs и CRISPR/ Cas, изменяя тем самым свойства растений (Хлесткина, Шумный, 2016). Системы ZFNs и TALENs не получили широкого распространения из-за сложности исполнения. Система геномного редактирования CRISPR/Cas наобо- рот оказалась достаточно простой и значительно способ- ствовала развитию геномного редактирования растений. Система CRISPR/Cas применяется на растениях более пяти лет. За это время она успешно апробирована на 24 культурах, для 16 из них, включая ячмень, получены модификации более 80 селекционно значимых генов (Короткова и др., 2017; Korotkova et al., 2019). Работы по редактированию генов ячменя ведутся преимущественно на модельном сорте Golden Promise. Редактированы не- сколько селекционно значимых генов ячменя. Так, в 2015 г. T. Lawrenson с коллегами (2015) сделали неработоспособными две копии гена HvPM19, кодирующего ABA-индуцируемый белок плазматической мембраны, что привело к сокращению продолжительности периода покоя семян. Полученные растения несут мутантные ко- пии гена-мишени, но не являются при этом трансгенными. В 2018 г. N. Kumar с коллегами (2018) нокаутировали ген MORC1 – негативный регулятор устойчивости к грибным патогенам, а S.V. Gerasimova с коллегами (2018a) полу- чили голозерные растения из пленчатых растений сорта Golden Promise, выведя из строя ген Nud1. Кроме того, при использовании метода трансфекции протопластов была впервые показана возможность успешной модификации генома немодельного сорта ячменя – сибирского сорта Алей (Gerasimova et al., 2018b).

Таким образом, cуществующая на сегодняшний день информация о потенциальных генах-мишенях и качество полногеномной последовательности ячменя представляют хорошую базу для применения технологий геномного редактирования с целью создания исходного материала для селекции сортов с заданными свойствами. Лимитирующим фактором для развития этого направления служит проблема получения растений-трансформантов других сортов ячменя, кроме модельного.

Изучение потомства от скрещивания сорта Golden Promise с сортами, обладающими низкой эффективностью трансформации, дало возможность выявить десять локу- сов, получивших наименования TFA1–TFA10 (transformation amenability). H. Hisano с коллегами (2017) представили способ получения генотипов, на которых в дальнейшем планируется проведение геномного редактирования, при этом в качестве донора генов эффективности трансформа- ции (TFA1, TFA2 и TFA3) предложено использовать Golden Promise.

## Заключение

Благодаря секвенированию генома ячменя, которое было завершено в 2012 г., произошла интенсификация гене- тических исследований, направленных на расшифровку механизмов формирования хозяйственно ценных призна- ков. Информация о последовательности генома оказалась полезной для широкого круга исследований. С помощью методов QTL и GWAS при применении SNP- и GBS-генотипирования выявлен ряд новых геномных районов, сцепленных с хозяйственно ценными признаками. В на- стоящее время результаты секвенирования в совокупности с технологиями высокопроизводительного генотипирова- ния можно использовать для эффективного направленного отбора нужных генотипов среди селекционных линий, что существенно ускорит создание новых сортов ячменя с заданными характеристиками.

## Conflict of interest

The authors declare no conflict of interest.
